# *De novo* Drug Delivery Modalities for Treating Damaged Hearts: Current Challenges and Emerging Solutions

**DOI:** 10.3389/fcvm.2021.742315

**Published:** 2021-09-28

**Authors:** Syed Baseeruddin Alvi, Salmman Ahmed, Divya Sridharan, Zahra Naseer, Nooruddin Pracha, Henry Wang, Konstantinos Dean Boudoulas, Wuqiang Zhu, Nazish Sayed, Mahmood Khan

**Affiliations:** ^1^Department of Emergency Medicine, The Ohio State University Wexner Medical Center, Davis Heart and Lung Research Institute, College of Medicine, Columbus, OH, United States; ^2^Division of Cardiovascular Medicine, Department of Medicine, The Ohio State University Wexner Medical Center, Columbus, OH, United States; ^3^Department of Cardiovascular Diseases, Physiology and Biomedical Engineering, Mayo Clinic, Phoenix, AZ, United States; ^4^Division of Vascular Surgery, Department of Surgery, The Stanford Cardiovascular Institute, Stanford University, Stanford, CA, United States

**Keywords:** myocardial infarction, heart failure, drug delivery, nanoparticles, clinical trials

## Abstract

Cardiovascular disease (CVD) is the leading cause of mortality, resulting in approximately one-third of deaths worldwide. Among CVD, acute myocardial infarctions (MI) is the leading cause of death. Current treatment modalities for treating CVD have improved over the years, but the demand for new and innovative therapies has been on the rise. The field of nanomedicine and nanotechnology has opened a new paradigm for treating damaged hearts by providing improved drug delivery methods, specifically targeting injured areas of the myocardium. With the advent of innovative biomaterials, newer therapeutics such as growth factors, stem cells, and exosomes have been successfully delivered to the injured myocardial tissue, promoting improvement in cardiac function. This review focuses on three major drug delivery modalities: nanoparticles, microspheres, and hydrogels, and their potential for treating damaged hearts following an MI.

## Introduction

Cardiovascular disease (CVD) affects approximately 30 million adults in the United States of America each year, resulting on average in 647,000 deaths (1). Myocardial infarction (MI) is one of the most common causes of CVD due to an insufficient blood supply to the myocardium that may lead to cardiomyocyte injury and death, resulting in left ventricular (LV) dysfunction and subsequent heart failure (2, 3). Currently, treatment of heart failure with reduced ejection fraction consists of medical management with beta-blockers, renin-angiotensin-aldosterone system inhibitors, angiotensin receptor neprilysin inhibitor, and sodium-glucose co-transporter inhibitors. These agents have pleomorphic effects that improve survival and, in certain cases promote LV reverse remodeling. Additionally, researchers have shown promising results with anti-inflammatory agents that prevent pro-inflammatory cytokines like interleukin-1 (IL-1) that can ameliorate cardiac stress and avoid remodeling (4). However, these pharmacological agents have limited effects on cardiac repair and recovery. Therefore, cardiac resynchronization therapy, when indicated, should also be applied due to the beneficial effects on mortality and morbidity in select heart failure patients.

In certain cases of significant coronary artery disease, revascularization may be required by percutaneous coronary intervention and/or coronary artery bypass grafting (CABG) surgery (5) to restore sufficient blood supply to the myocardium. However, these interventions are limited by their inability to induce localized angiogenesis to injured tissue (6) and ability to rejuvenate damaged tissue. Therefore, exploring newer therapies for localized and controlled angiogenesis of the damaged cardiac tissues becomes essential for cardiac tissue repair and recovery.

**Figure 1:**
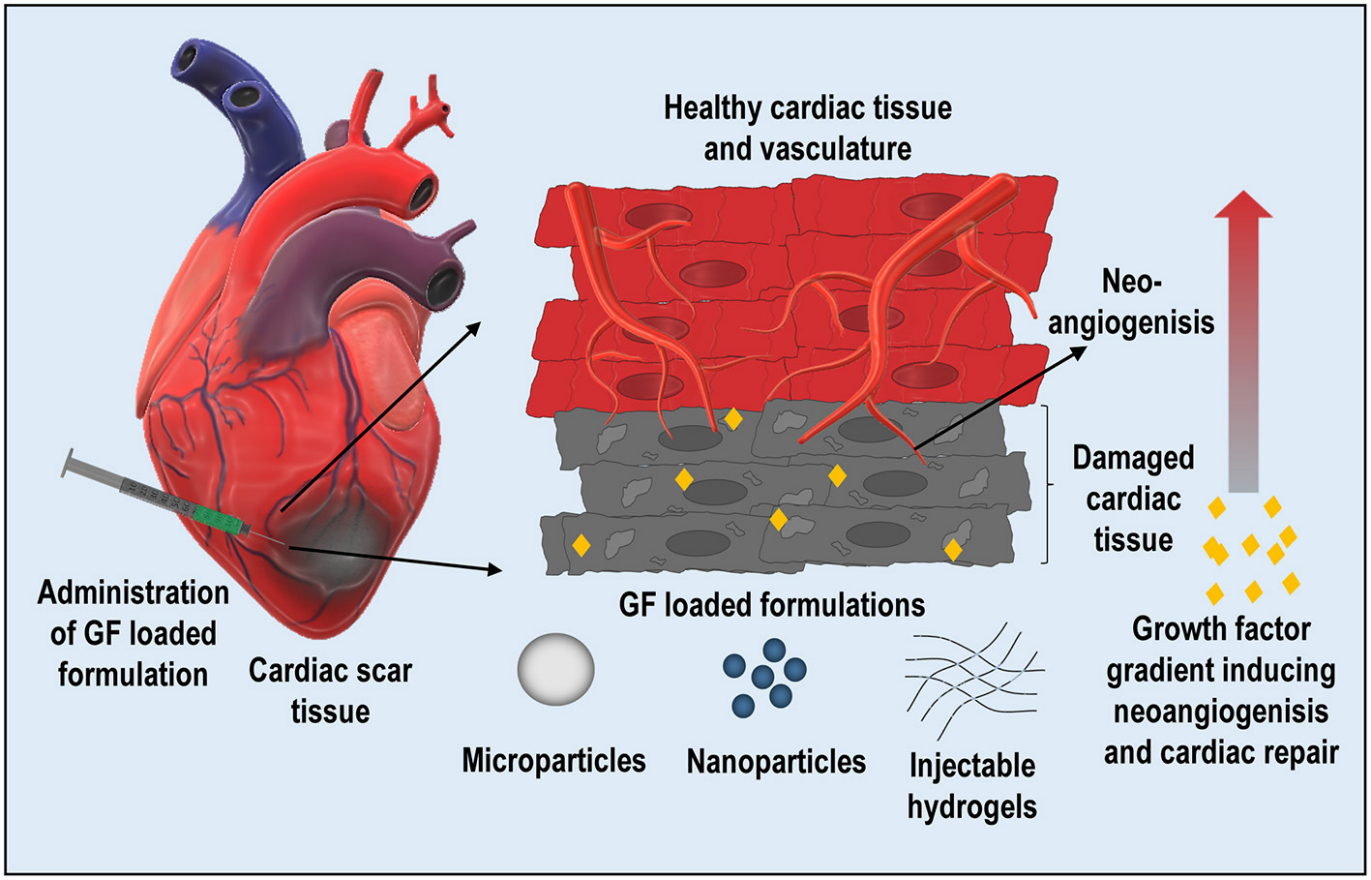
Schematic representing the intramyocardial delivery of growth factor-loaded formulations (nanoparticles, microspheres, and hydrogels) for localized and sustained delivery for repairing the damaged cardiac tissue.

Growth factors are bioactive molecules that exhibit a paracrine effect on efferent cells, like endothelial and fibroblasts regulating the extracellular matrix (ECM) deposition (7). There has been a growing interest in identifying and exploring novel recombinant growth factors as possible candidates for tissue repair and regeneration (8, 9). However, clinical trials conducted on the efficacy of these molecules failed to produce clinically relevant results (10–12). Despite the bioactivity of growth factors, its clinical efficacy was not as expected due to its poor stability, biological half-life, target specificity, among others. This warrants newer formulations with enhanced stability and desired pharmacokinetic profile to achieve clinically relevant therapeutic outcomes. This review highlights the recent advances and strategies adopted to administer growth factors utilizing nanoparticles (NPs), microparticles (MPs), and hydrogels for cardiac repair (Figure 1) and relevant clinical studies.

## Fabrication of Nanoparticles and Microspheres

Nanoparticles can be fabricated by various methods depending on the type of polymer and drug used. Most NPs used for drug delivery purposes are based on lipids or polymeric nanosystems such as DSPC, HSPC, PLA, and PLGA. These nanoparticles can be prepared by thin-film hydration techniques, solvent evaporation, and co-precipitation, respectively. However, more extensive fabrication techniques have been reviewed elsewhere (13–19). A variety of hydrophobic and hydrophilic therapeutics molecules can be encapsulated in these nanoparticles by the single or double emulsification method, respectively (20–23). In general, the hydrophobic drugs and polymers are dissolved in solvents like chloroform, diethyl ether to form a non-aqueous phase. To this, an aqueous phase containing a stabilizer is added and emulsified; later, the emulsion is subjected to sonication for size reduction and allowed to stir for solvent evaporation (24–28). The single emulsion evaporation method yields an excellent encapsulation of hydrophobic drugs. However, the therapeutic proteins that are used for cardiac regeneration are water-soluble and exhibit poor stability, thus encapsulating them into a nanosystem involves additional excipients like heparin, and albumin, trehalose (29–31). More importantly, the double emulsification method for encapsulation of therapeutic proteins exhibits higher encapsulation and enhanced stability of the nanosystem.

The microspheres can be fabricated by following similar techniques and using similar polymers; however, factors like homogenization speed, sonication time, the molecular weight of the polymers can significantly influence the size and homogeneity of the microspheres (32–35). It has been observed that despite optimizing these variables, the exquisite tuning of the microsphere's size has offered significant challenges. It is of great significance to obtain uniform microspheres, especially for cardiac application, as microsphere embolism can lead to micronecrosis in cardiac tissues (36). More recently, microfluidic-based approaches are gaining attention to prepare microspheres with a high degree of uniformity and size tunability (37, 38). However, large-scale preparation of the microspheres is one of the significant challenges of using microfluidics (37). These drug delivery systems can be characterized for the shape and size by employing scanning electron microscopy (SEM), transmission electron microscopy (TEM), dynamic light scattering (DLS), and nanoparticle tracking analysis (NTA).

## Fabrication of Hydrogels

Hydrogels are extensively employed for the purpose of drug delivery. In general, hydrogels are fabricated by crosslinking hydrophilic polymers forming microporous three-dimensional structures. The porosity of hydrogels can be controlled by changing the degree of crosslinking, thereby influencing the rate of drug release. More importantly, hydrogels can entrap hydrophilic therapeutic proteins with high efficiency and exhibit sustained release as needed. A variety of hydrogels have been reported in the literature for various applications ranging from drug delivery, stem cell delivery, scaffolds to implants. Fabrication techniques for these hydrogels have been discussed elsewhere with extensive details. However, this review highlights the fabrication of a few smart hydrogels employed for cardiovascular applications.

Hydrogels have been broadly categorized into two, i.e., hydrogels derived from natural sources like polysaccharides, proteins, and hydrogels derived from synthetic sources like polyethylene glycol (PEG). These hydrogels can further be modified by conjugating molecules on the activated functional groups present on the polymer by click-chemistry approaches. Functionalization of these polymers imparts newer functionalities to the hydrogel, like pH or temperature responsiveness, thus rendering them smart hydrogels. Researchers have used naturally occurring alginate that exhibits properties like shear thinning, excellent biocompatibility, and the ability to encapsulate living cells for cardiovascular and wound healing applications. The –OH group present on the alginate is oxidized by using oxidizing agents like sodium peroxide leading to the formation of alginate dialdehyde (39, 40). The aldehyde functional groups on alginate have been crosslinked with –NH_2_ groups present on gelatin, forming self-crosslinked hydrogels to deliver growth factors and stem cells. Similarly, gellan gum, a naturally occurring polysaccharide, has been used to fabricate biomimetic scaffolds for cardiac cells. The hydrogel scaffold was first fabricated by oxidizing –OH groups present on gellan by sodium periodate, leading to aldehyde functionalized gellan. Later, the modified gellan was crosslinked with gelatin forming hydrogel scaffold (41, 42). In a similar line, chitosan has also been used as a thermosensitive polymer for various tissue engineering applications (43). Biocompatible crosslinkers like tri-polyphosphate and β-glycerophosphate can easily crosslink and impart temperature responsiveness rendering them injectable (44, 45). Furthermore, self-assembling peptide-based injectable hydrogels have demonstrated immense potential for clinical application. Briefly, these self-assembling peptides with repeating units like “AEAEAKAKAEAEAKAK” can be synthesized using a solid-phase peptide synthesis technique on a substrate (46, 47).

Synthetic polymer-based hydrogels display versatile properties with a high degree of tunability. One of the most commonly used polymers for tissue engineering and drug delivery applications is polyethylene glycol (PEG) (48). The functionalization of PEG imparts newer properties to the hydrogel, and the most commonly used is methacrylate derivitization. Polyethylene glycol diacrylate (PEGDA) is a biocompatible, light-responsive polymer that undergoes crosslinking in the presence of a radical initiator (49). The crosslinking is facilitated by the UV/visible light irradiation, and the so formed hydrogel exerts mechanical stability, which can be used for encapsulating cells. PEGDA can be synthesized by a one-pot microwave-assisted technique where methacrylic anhydride is mixed with PEG and subjected to microwave irradiation for 5 min, resulting in PEGDA (50). Similarly, polyvinyl alcohol (PVA) has also been functionalized with glycidyl methacrylate (GMA) to impart photocrosslinking of PVA. The –OH group on PVA was reacted with GMA in the presence of sulfuric acid at elevated temperature (80–100°C); the resultant polymer was precipitated in acetone (51).

## Nanoparticles Mediated Delivery

To overcome the challenges faced by conventional drug delivery, nanoparticle-based formulations have been evolved. Nanoparticle formulations can encapsulate the therapeutic cargo and deliver it to the specific tissue, which gives NPs an edge over the conventional formulation. In addition, recent advancements in nanotechnology have led to developing newer materials for tissue-specific delivery ranging from organic (polymers, lipids, proteins, among others) to inorganic (gold, iron, silica, among others) materials (52, 53). These nanosystems have shown enormous potential in treating cancers due to their ability to target tissues. However, in recent times they are also being explored in treating other diseases.

For the delivery of bioactive molecules to damaged cardiac tissue, a substantial amount of research has focused on developing nanoparticles that can precisely deliver the therapeutics to the targeted tissue, as shown in Table 1. One of the main reasons for adopting nanoparticles for drug delivery is the size of the material and the ability to target specific tissues by employing site-specific ligands. With the ease of surface functionalization and click chemistry, various targeting ligands can be grafted onto the NPs that limit the off-target toxicity. One successful strategy to deliver the therapeutics to the damaged myocardium is by targeting the damaged extracellular matrix (ECM). Huang et al. have reported a polylactic acid (PLA) conjugated with a shot peptide (cys–arg–glu–lys–ala, a clot binding peptide) to deliver thymosin beta 4 (Tβ4) for cardiac repair. This study investigated the possibility of targeting fibrin deposited in the damaged myocardium to deliver therapeutic peptides effectively. The results demonstrated sustained retention of the NPs within the injured myocardium for 7 days post intravenous (IV) administration and a significant reduction in scar tissue (63).

**Table 1:** Studies highlighting the nanoparticle based formulations delivering therapeutics like miRNA, proteins and small molecules for cardiac repair.

**Authors**	**Therapeutics**	**Materials**	**Outcomes**	**References**
Liu et al.	Exosomes	Fe3O4 core, silica shell, polyethylene glycol	Angiogenesis and improved heart function in the infarcted region of heart	(54)
Krohn-Grimberghe et al.	siRNA	Polyethylene lipid conjugates	Decrease leukocytes, improved healing, and prevented heart failure in diseased heart	(55)
O'Dwyer et al.	Stromal-Derived Factor 1α (SDF)	Poly(glutamic acid) (PGA) polypeptides	Gap closure on the scratch assay, increase in tubule length on Matrigel assay	(56)
Mohtavinejad et al.	Diagnosing myocardial infarctions at early stages	Polyethylene glycol diacid	Diagnosing a myocardial infarction at an early stage at a low cost and low toxicity	(57)
Sayed et al.	miRNAs	G poly(amidoamine)-histidine (PAMAM-His)	The antiapoptotic effect, prevented hypoxia/reperfusion-induced apoptosis	(58)
Li et al.	Peurarin	Triphenylphosphonium (TPP) cation	Decrease in apoptotic cells and ROS levels. Micelles can also be targeted to ischemic region	(59)
Guo et al.	Tanshinone and Puerarin	Methoxy polyethylene glycol	Cardioprotective mechanisms	(60)
Chen et al.	Stem Cell Therapy	Silica-iron oxide	Increase in LV ejection fraction, improvement in stem cell survival	(61)
Yokoyama et al.	Adipose-derived stem cell therapy	Poly(lactic-co-glycolic) acid PLGA	Cardiac regeneration after a myocardial infarction	(62)

As the nanoparticle's size decreases, the effective surface area that can be used for grafting the molecules of interest increases substantially. Chang et al. reported the effect of poly(lactic-co-glycolic acid) (PLGA) NP's size on payload retention. Insulin-like growth factor (IGF-1) was employed for its cardioprotective effect. The results demonstrated that 60 nm PLGA-NPs exhibited a higher loading efficiency and were successfully retained at the injected site for 24 h (h) post-intramyocardial delivery. The *in vivo* study showed inhibition of scar formation and ventricular remodeling, thus displaying the cardioprotective nature of these NPs (64).

Following an acute MI, the inflammatory response leads to the recruitment of pro-inflammatory macrophages and myofibroblasts at the infarcted site; this results in scar formation, leading to LV dysfunction. Nanoparticle-based therapies targeting and modulating these inflammatory cells' responses can present a promising approach for preventing scar formation. Tokutome et al. have reported PLGA-NPs entrapping pioglitazone for tuning the localized immune response by IV administration following a MI. This study's rationale was to inhibit the activation of macrophages to the M1 phenotype (pro-inflammatory). When entrapped in PLGA-NPs, pioglitazone was selectively delivered to the macrophages (both local and splenic) and prevented the activation of macrophages, thus providing an anti-inflammatory response. The results demonstrated enhanced LV functioning and minimized scar formation. Furthermore, PLGA and pioglitazone's use imparts a more significant advantage as they are Food and Drug Administration approved and holds immense potential for clinical translation (65).

Nanoparticle-based therapies have also been explored for inducing angiogenesis; Ruvinov et al. reported polyelectrolyte complex NPs of cationic angiogenic growth factors complexed with anionic alginate sulfate. These nanoparticles showed significant improvement in LV functioning and superior angiogenesis (66). Quadros et al. also reported PLGA-NPs encapsulating adrenomedullin-2 (ADM-2), a proangiogenic and cardioprotective peptide. A sustained release was observed lasting for ~21 days while inducing endothelial cell growth when tested *in vitro*. These reports demonstrated the feasibility of using NPs for collateral angiogenesis in the damaged myocardium (67). Izadifar et al., reported a hybrid lipid-based nanoparticle encapsulating a cocktail of growth factors containing platelet-derived growth factor (PDGF), vascular endothelial growth factor (VEGF), and basic fibroblast growth factor (bFGF). The bilayered nanoparticle encapsulating the growth factors exhibited a controlled release that could be employed for cardiac regeneration (68). Furthermore, Imanishi et al. have used morphogen sonic hedgehog (Shh) that has a great potential for tissue repair and regeneration post-MI, but the poor pharmacokinetic profile limits its applicability. To address this, a polyelectrolyte complex (PEC) was formulated that could encapsulate Shh and could be used for safe delivery to the myocardium. The study demonstrated a sustained release for up to 3 weeks resulting in the repair of cardiomyocytes (69).

**Figure 2:**
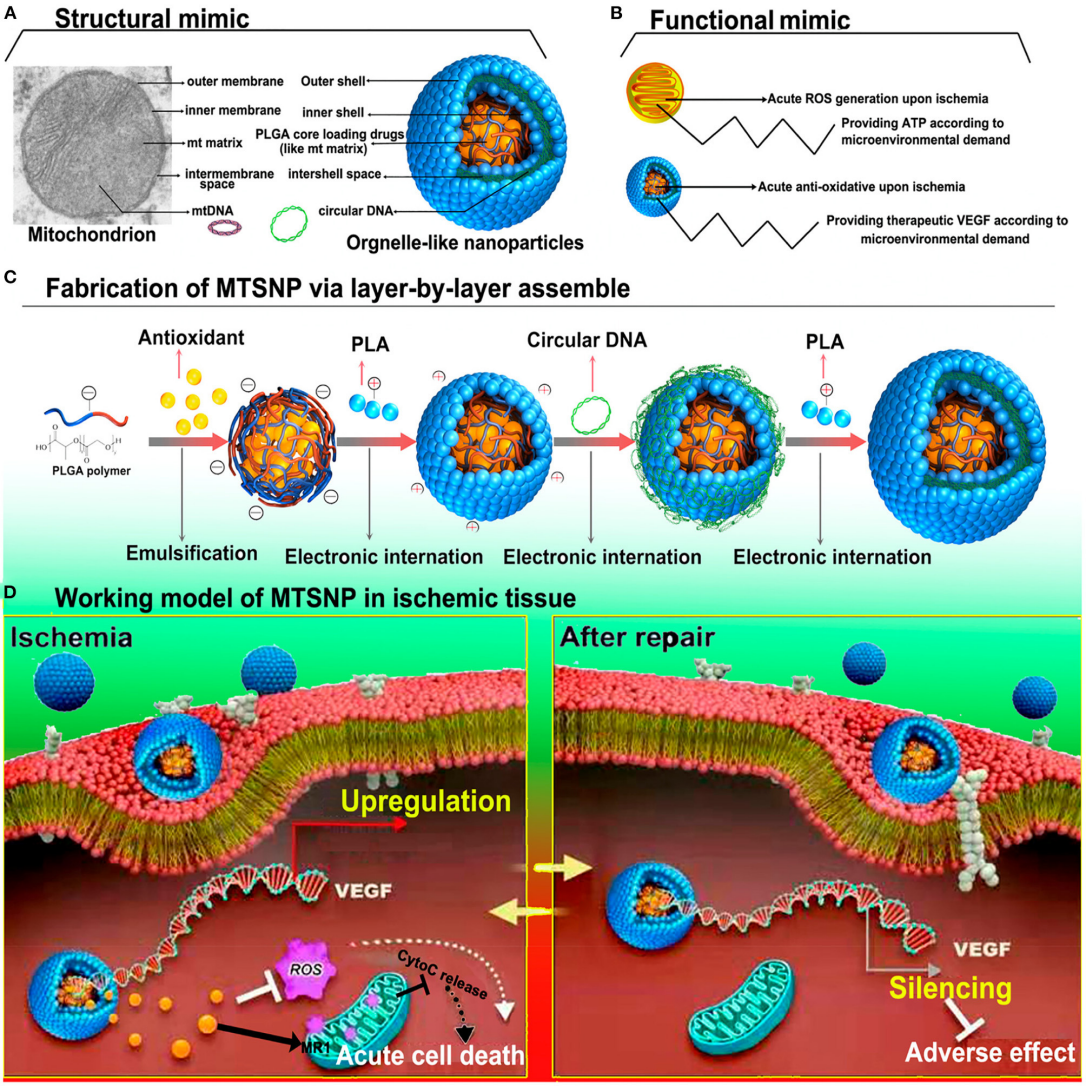
Schematics representing the fabrication of mitochondria mimicking smart polyelectrolyte nanoparticle and their mechanism of action. **(A)** Structural mimic of organelle-like nanoparticle, **(B)** Functional similarity of the mitochondrion and organelle-like nanoparticle. **(C)** Fabrication of organelle-like nanoparticles by layer-by-layer approach. **(D)** Ischemia responsive activation of VEGF expression facilitated organelle-like nanoparticle and their antioxidant potential (70).

Nanoparticles have also been used for gene transfection *in vivo;* the nonmetric size enhances cell penetration and transfection. As the genetic material exhibits a strong negative charge, it is often complexed with the positively charged NPs. Lin et al. have reported a smart hybrid system whose core was comprised of an antioxidant and was further coated with positively charged polymer in a layer-by-layer fashion. The surface of the nanoparticles were then complexed with plasmid DNA. The rationale of the study was combining the cytoprotective effect of melatonin (present in the core) along with hypoxia/normoxia responsive VEGF plasmid DNA (Figure 2). The smart NPs presented as an environment-responsive nanosystem that triggers VEGF in hypoxic conditions make it a viable system for both acute and chronic cardiac repair (70). Additionally, Ye et al. reported a similar hypoxia-responsive VEGF plasmid complexed with polyethyleneimine (PEI) for the transfection of skeletal myoblast. These NPs exhibited superior transfection efficacy, and the transfected cells showed enhanced VEGF expression under hypoxic conditions. When tested *in vivo*, a significant enhancement in LV functioning was observed (71).

One of the primary factors for myocardial cell death during ischemia is reperfusion injury. The first-line therapy for alleviating the myocardial ischemia is to perform reperfusion. However, an increase in intracellular reactive oxygen species (ROS) resulting from reperfusion surpasses the normal cell's antioxidant mechanism, thus causing cell death. To prevent ischemic injury, attempts have been made to fabricate enzymes mimicking NPs as a possible route to myocardial salvage. Zhang et al. fabricated a biomimetic artificial nanozyme that mimics the activities of enzymes like superoxide dismutase (SOD), catalase (CAT), and peroxidase (POD). These nanozymes target mitochondria by triphenylphosphine (TPP) and result in a significant quenching of ROS in the ischemic region following IV administration with subsequent improvement in LV functioning (72).

## Microsphere Based Delivery

Microspheres bring a whole different paradigm to the world of drug delivery due to their relatively small size and other unique properties such as improved drug release kinetics and stability. As microspheres hold a larger volume than NPs, they can encapsulate various drugs and exhibit sustained release. Different polymers have been explored for the fabrication of MPs, including natural (alginate, chitosan, starch, among others) and synthetic (PLGA, Polycaprolactone (PCL), PLA, among others) (Table 2). They can encapsulate small molecules, therapeutic peptides, and proteins and have been explored as a therapeutic modality for myocardial repair. In order to exert a therapeutic effect, the microparticles must retain within the myocardium for an extended period of time, enabling a sustained and localized delivery of bioactive materials. In a similar line, Formiga et al. has investigated the retention of PLGA based MPs with varying sizes (2–30 μm); among these various sizes, 5 μm showed the highest compatibility and myocardial retention for up to 3 months post intramyocardial delivery suggesting the use of MPs for myocardial regeneration (79).

**Table 2:** Microparticle based formulations used as a depot for sustained delivery of growth factors aiding in cardiac repair.

**Authors**	**Therapeutics**	**Materials**	**Outcomes**	**References**
Nie et al.	Nicotinamide riboside	Drug co-crystals	Protection against acute heart injury with no cytotoxic effects on any major organs	(73)
Feng et al.	Insulin-like growth factor 1	Silk fibroin microspheres	Reduction of infarct size and overall improvement of cardiac function	(74)
Zhang et al.	Endothelial growth factor	poly(lactic-co-glycolic acid) (PLGA)	Enhanced proliferation of endothelial cells and promoted capillary and smooth muscle formation	(75)
Song et al.	Embryonic stem cells	Poly-ε-caprolactone (PCL)	Microspheres aided in embryonic stem cell differentiation which can be used for delivery to the myocardium	(76)
Rodness et al.	Vascular endothelial growth factor (VEGF)	Calcium-alginate	Improved cardiac function when compared to control, but resulted in thicker scars with high capillary density	(77)
Arunkumar et al.	Basic Fibroblast Growth Factor (bFGF)	PCL	Rapid angiogenesis in gel plug assay	(78)

The acute inflammatory response followed by MI often results in LV remodeling, and attempts have been made to inhibit this inflammatory response as a possible treatment. Pro-inflammatory cytokines like IL-β are released following myocardial cell damage. Thus, IL-β inhibitors potentially can serve as a therapeutic target; however, IL-β inhibitors, due to their systemic inhibition of IL-β, results in fatal adverse events. In an attempt to mitigate adverse systemic effects, they have been delivered locally using MPs. Li et al. reported a platelet mimicking MPs grafted with anti-IL-1β for a localized anti-inflammatory response. The study's rationale was to target the infarcted region by platelet-derived membranes that were grafted with anti-IL-1β. The *in vivo* results indicated specific localization of the microparticles within 8 h of injection, which lasted for 72 h. It showed a sustained targeting ability of the microsystem essential to inhibit the acute inflammatory response following an MI. Moreover, the same microparticles showed no accumulation in cardiac tissue when injected in normal mice, further proving its targeting efficacy during MI (80).

**Figure 3:**
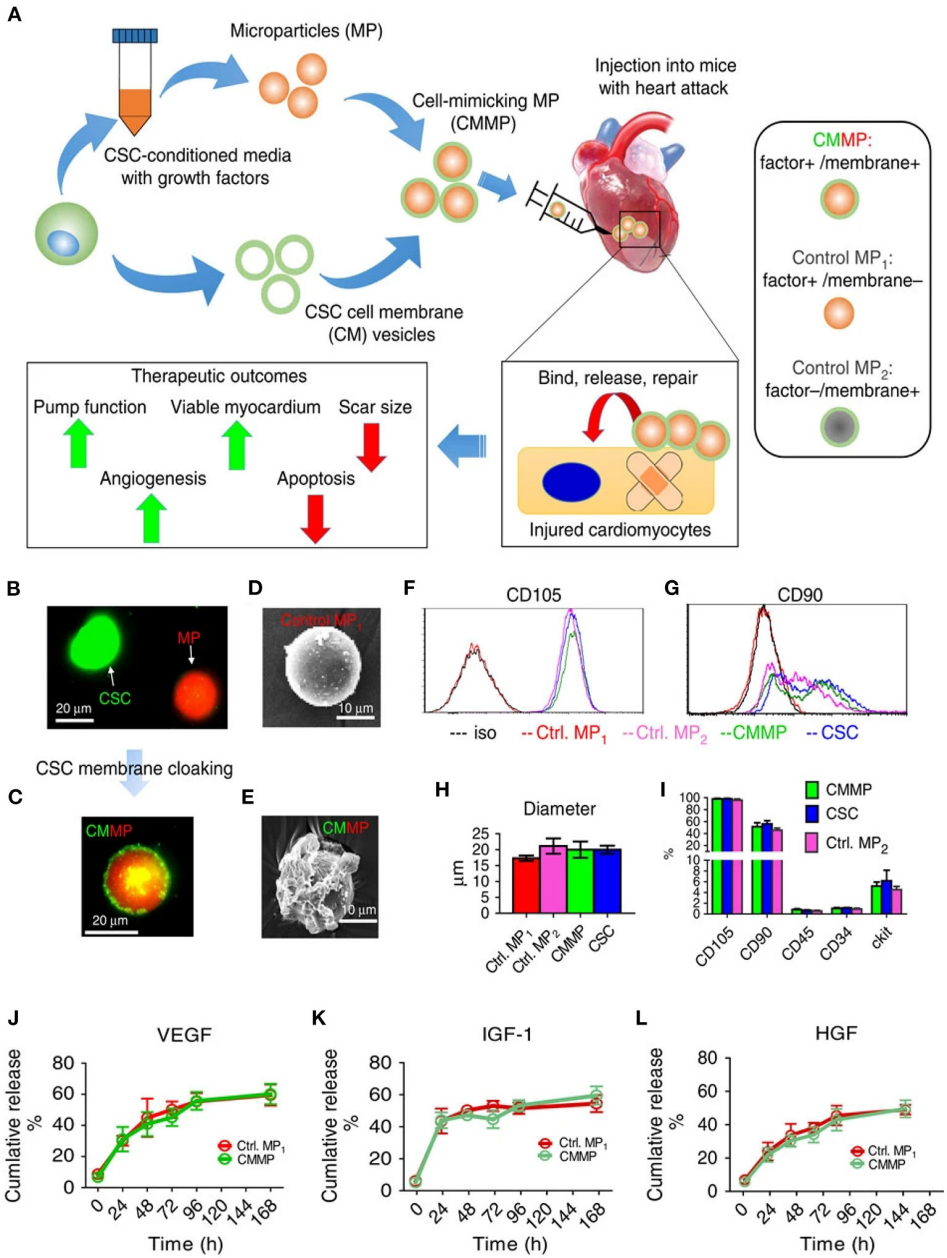
**(A)** Schematic representing cardiac stem cell (CSC) mimicking microspheres fabricated by coassembly of CSC-derived membranes on polymeric microspheres. **(B,C)** Fluorescence imaging of CSC (green) and microsphere (red) tagged with fluorescent dyes **(D,E)** SEM of blank microsphere and CSC coated microsphere. **(F,G)** Evaluation of CSC markers on fabricated hybrid microspheres. **(H)** Size analysis of hybrid microspheres and respective controls. **(I)** CSC antigen analysis of hybrid microspheres. **(J–L)** VEGF, IGF-1, and HGF growth factors release profiles from the hybrid microspheres, respectively (81).

Cardiac stem cells mimicking MPs were reported by Tang et al., and the rationale of this study was to mimic the paracrine and cell-cell adhesion properties of stem cells. This was achieved by fabricating a PLGA core entrapping growth factors like VEGF, insulin-like growth factor 1 (IGF-1), and hepatocyte growth factor (HGF) that were released in a sustained manner (Figure 3). The same growth factor releasing cargo was coated with stem cell-derived membranes, mimicking both paracrine factors (growth factors) and cell-cell interaction (by membrane coating) of stem cells. Being an acellular approach, one of the major advantages of this is the lack of immune rejection, which is otherwise evident in stem cell implantation. Moreover, when injected intramyocardially following a MI, a reduction in scar size and enhanced angiomyogenesis was observed (81). This presents a newer acellular approach for remuscularization of infarcted myocardium as a possible therapeutic strategy.

The inflammatory response following MI is induced by infiltrating pro-inflammatory macrophages within the infarcted region. It has been reported that macrophage polarization toward a reparative phenotype could prevent inflammation and repair the damaged tissues. Pascual-Gil et al. used polymer-based MPs as a way to achieve sustained and local delivery, specifically to the damaged heart focusing on the relationship between neuregulin-1(NRG-1) loaded PLGA-MPs and inflammation. They discovered that the intramyocardial delivery of NRG-1 PLGA-MPs improved the immune response post-MI. Interestingly, when these MPs were administered at various time points, they were shown to improve the anti-inflammatory response post-MI (82).

NRG-1 and fibroblast growth factors (FGF) typically have a shorter half-life and do not have a specified target. However, the pharmacokinetics of both of these growth factors can be significantly improved when used as MPs leading to enhanced bioavailability. Therefore, researchers have increasingly used MPs to deliver these growth factors to treat the infarcted regions post-MI. Similarly, Formiga et al. have used the fibroblast growth factor-1 (FGF1) and NRG-1 growth factors encapsulated in PLGA-MPs and administered to the heart after an acute MI. Additionally, they were able to achieve a sustained release of the growth factors for a more extended period. Three months after intramyocardial administration of MPs, a significant reduction in infarct size was seen with a lower rate of fibrosis than control (83) and improvement in overall cardiac function.

Microparticles have also been explored for the localized delivery of stem cells and engraftment, as reported by Savi et al. The MPs encapsulating hepatocyte growth factor (HGF) and insulin-like growth factor 1 (IGF-1) were fabricated and complexed with adipose-derived stem cells. They successfully formed a substrate for the cell attachment and, in addition to it, acted as a reservoir for sustained release of growth factors. When these cells and MPs complexes were administered into mice models, they exhibited localized stem cell retention that stimulated the healing of the infarcted myocardium and restored hemodynamics within the region. However, there is an unmet need to address the arrhythmias arising from cell engraftments (84). Additionally, Zhang et al. reported a combination of HGF with IGF-1 and its protective effect on implanted bone marrow mesenchymal stem cells (BMSCs) as a treatment modality for the damaged heart. When injected into the damaged myocardium, the growth factor-loaded MPs showed reduced apoptosis and enhanced myocardial differentiation resulting in improved LV function (85).

Madonna et al. have employed fibronectin-coated PLGA-MPs encapsulating VEGF as a carrier for adipose tissue-mesenchymal stromal cells. These microcarriers were injected into the infarcted region of the heart. Similarly, treatment with these MPs demonstrated improvement in LV function (86). In a similar study, Rosellini et al. reported biomimetic gellan/gelatin microspheres loaded with IGF-1 combined with cardiac progenitor cells for localized cell engraftment and sustained release of growth factors. The results demonstrated cardiac progenitor cell adhesion to the MP's surface. These microspheres also reduced myocardial damage and improved overall cardiac function and successful cell engraftment (87).

Following an MI, a drastic increase in the ROS levels is observed in both intracellular and extracellular environments. Thus, enriching the environment with antioxidant enzymes could be used as a therapeutic strategy. Seshadri et al. reported polyketal MPs encapsulating SOD1 quenching ROS both in the intracellular and extracellular environment. On the contrary, only extracellular ROS was quenched when the free enzyme was administered. Furthermore, once injected intramyocardially, the MPs were retained for 3 days in the myocardium and prevented myocardial cell death (88).

## Hydrogels Mediated Delivery

Hydrogels are one of the most extensively researched therapeutics for drug delivery and tissue engineering. They are composed of a crosslinked polymer network by forming covalent bonds, hydrogen bonds, and hydrophobic interactions between the polymer chains, thereby forming a depot (89). The use of biocompatible and biodegradable polymers to form a 3D matrix accommodating cells, drugs, NPs, MPs, or combinations has further expanded the applicability of hydrogels. A variety of hydrogels have been reported as a modality for repairing the injured myocardium (Table 3). However, the primary considerations in designing hydrogel for cardiac repair are its injectability and stable depot formation at the injected site. Significant improvements have been made in the design and materials for the hydrogels, which respond to stimuli like temperature, light, enzymes, pH, among others. Some of the recent advancements in the field for myocardial repair are discussed in the following section.

**Table 3:** Studies highlighting the hydrogel formulations used to delivering cells, miRNA, small molecules and growth factors for cardiac repair.

**Authors**	**Therapeutics**	**Materials**	**Outcomes**	**References**
Shvartz et al.	Amiodarone	Sodium alginate	Prevented postoperative atrial fibrillation post coronary artery bypass surgery.	(90)
Zhu et al.	Induced pluripotent stem cells	Extracellular matrix hydrogel	Robust cardiovascular repair post-MI	(91)
Malektaj et al.	Niosomal rosuvastatin	Poly (N-isopropyl acrylamide) (PNIPAAm)	Enhanced angiogenesis	(92)
Radmanesh et al.	microRNAs	4 arm PEG in combination with polyelectrolyte complex	Enhanced angiogenesis and enhancement of capillary density	(93)
Contessotto et al.	Elastin-like recombinamers	Extracellular matrix hydrogel	Reduced fibrosis and more angiogenesis occurring as well as preservation of cardiomyocytes	(94)
Sim et al.	Bone marrow-derived mesenchymal stem cells	Heart derived extracellular matrix hydrogel	Improvement in cardiac function and protection of myocardium	(95)
Lyu et al.	Human mesenchymal stem cells	Hyaluronic acid hydrogel	Decreased inflammatory response and revascularization of the infarct region	(96)
You et al.	Mesenchymal stromal cells	Poly(2-alkyl-2-oxazoline) (POx) derivative, based on 2-ethyl-2-oxazoline and 2-butenyl-2-oxazoline	Fibrosis reduction and improvement of overall cardiac function as well as neovascular formation	(97)
Ding et al.	ROS scavenging and O_2_ generating gel	3 s hyperbranched polymers and methacrylate hyaluronic acid (HA-MA)	Removal of excess ROS, reduction in infarct size, inhibition of apoptotic cells	(98)

The remodeling of the LV following an MI is the consequence of a cascade of inflammatory responses that activates ECM degrading enzymes. Attempts have been made to inhibit the activity of such enzymes from preventing cardiac remodeling after MI. Awada et al. has fabricated fibrin-based hydrogel entrapping polyelectrolyte complexes for myocardial repair. The rationale of the study was to avoid remodeling and to remuscularize the injured myocardium. To achieve this, metalloproteinases-3 inhibitor (TIMP-3) was entrapped in the fibrin matrix for rapid release and inhibition of ECM degradation. Secondly, for remuscularization, FGF-2 and stromal cell-derived factor 1 (SDF-1α) were formed into polyelectrolyte complexes for sustained release. Taken together, the timely release of these proteins from hydrogel showed significant improvement in cardiac functionality up to 8 weeks post-treatment (99).

The paracrine factors released from the stem cells have shown significant improvements in cardiac function. Tang et al. reported P(NIPAM-AA) based nanogels encapsulating cardiac stem cells for localized delivery to the myocardium. The stem cell embedded nanogels were injected in the immune-competent mice that showed no inflammatory immune response prolonging its retention, which otherwise was not observed with only stem cell injections. Results demonstrated a significant improvement in cardiac functionality and remuscularization when compared with only stem cell injections. More interestingly, the group treated with the only nanogel also showed considerable improvement, including scar inhibition, which could have been due to the formation of a physical barrier for immune cell infiltration suggesting the beneficial role of hydrogel (100). Among similar lines, Kanda et al. reported cocoon-like nanoporous gels (NPGs) and studied the effect of the varying NPG content. They found that by increasing NPG concentration, significant enhancement in cell migration and cytokine and extracellular vesicles (EV) secretion were observed. Taken together, these NPG's when administered *in vivo* were found to improve cardiac function and boosted the generation of new blood vessels (101).

Furthermore, Fang et al. have shown the potential of sustained delivery of 6-Bromoindirubin-3-oxime (BIO) and IGF-1 in a hybrid hydrogel system, which could help induce cardiac repair *in vivo*. The use of small molecules like BIO that promotes cell cycles in adult cardiomyocytes can aid in remuscularization. Both the BIO and IGF-1 were encapsulated within gelatin NPs. The results demonstrated that the hybrid gel material, when administered to MI-induced rats, showed proliferation of cardiac cells, enhanced revascularization around the infarcted area, and improved overall LV function (40).

The miRNA has shown promise in treating various diseases, including CVD. The limiting factor for miRNA-based therapies is the lack of cell internalization and delivery vehicles. Along similar lines, Yang et al. reported a poly(9,9-dioctylfluorene-alt-benzothiadiazole) (PFBT) based miRNA delivery system. These NPs were loaded within a hydrogel matrix and administered intramyocardially. The miRNA was seen to trigger the proliferation of human embryonic stem cell-derived cardiomyocytes and endothelial cells (hESC-CMs and hESC-ECs), and promote angiogenesis during hypoxia with lower toxicity than other treatments. Following hydrogel injection, LV function was improved by increasing the ejection fraction from 45–64% and reducing scar size from 20–10% while doubling the capillary density. This introduced a mechanism to deliver miRNA to restore LV function (102).

**Figure 4:**
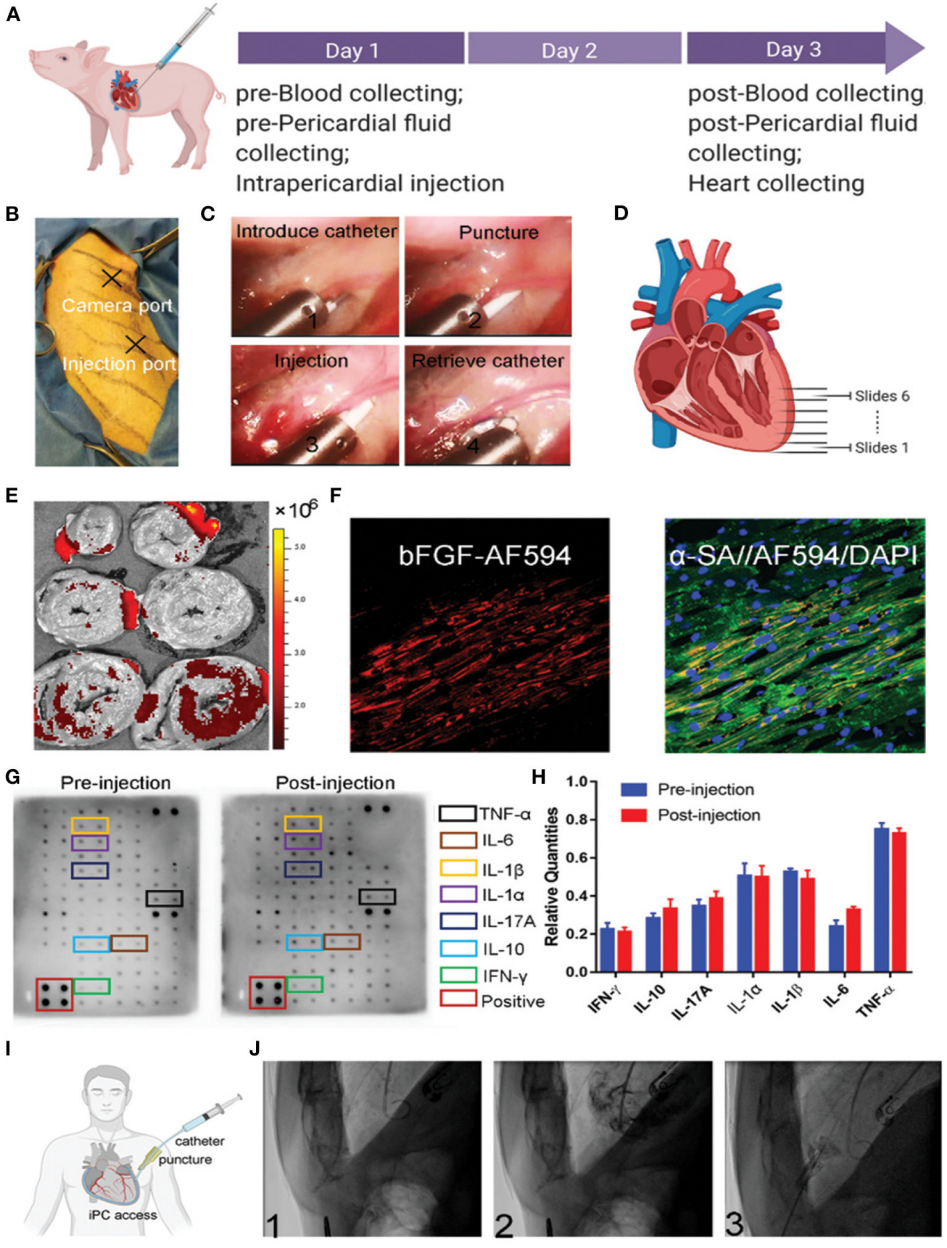
**(A)** Schematics representing the protocol for large animal studies for smart hydrogel. **(B)** Photograph showing the position of the camera and an injection port. **(C)** Sequential representative images are displaying the administration of smart hydrogel. **(D)** Schematic representing the sectioning of the isolated heart. **(E)** *Ex vivo* NIR imaging of cardiac slices demonstrating the accumulation of injected smart hydrogel. **(F)** Fluorescence imaging of bFGF. **(G,H)** Quantitative and qualitative analysis of cytokines before and after administration of smart hydrogel. **(I,J)** The feasibility of minimally invasive catheter-based administration into the pericardial cavity in a human subject (103).

Cardioprotective hydrogel based on crosslinked polyvinyl alcohol (PVA) has been reported by Li et al. The gel undergoes degradation when exposed to ROS. The advantage of using ROS-responsive hydrogels is that they can prevent ischemic reperfusion injury, where a burst of ROS is produced in response to reperfusion. More interestingly, the hydrogel can also release encapsulated cargo, like bFGF, in response to ROS. Furthermore, the authors have also demonstrated the feasibility of administering the hydrogel to the pericardial cavity in large animals and humans (Figure 4), resulting in significant improvement in LV function and enhanced angiogenesis (103).

The peptide-based hydrogels have opened up a newer array of biocompatible, self-assembled, and biodegradable materials. The primary advantage of a peptide hydrogel over others is the ability to tune the mechanical and chemical properties by altering the amino acid sequences. For example, Lin et al. reported a peptide hydrogel and employed it as a cell delivery material for cardiac repair. When the gel was injected into the myocardium of pigs following an acute MI resulting in heart failure, LV function was improved. More interestingly, the peptide gel alone was also able to show improvement in cardiac function. These findings agree with earlier reports suggesting that blank hydrogels themselves can impart therapeutic benefit and aid in repairing injured myocardium (104).

Similarly, Bai et al. combined bioactive ECM hydrogels and high cardiomyogenic seeding cells for cardiac repair. These hydrogels were shown to support proliferation and cardiomyogenic differentiation, all while improving overall cardiac function. These results were observed both *in vitro* and *in vivo* (105). Lastly, Dong et al. developed a conductive hydrogel that provides an excellent vehicle for cell delivery post-MI. This hydrogel also exhibited self-healing behavior and enhanced cardiac cell viability when entrapped in the gel matrix (106).

## Clinical Trials

A growing body of literature shows that a relatively large number of therapeutic modalities are being taken up for clinical testing for various illnesses like CVD, cancer, and wound healing. The clinical translation of newer research modalities is often challenged with stringent criteria to demonstrate the safety and efficacy of the formulations. However, there is a need to bridge the gap from bench to bedside, enabling researchers to develop clinically relevant and translatable therapeutics. In the following section, recent clinical studies conducted for cardiac theranostics involving NPs, MPs, and hydrogels (summarized in Table 4) are discussed.

**Table 4:** Clinical studies conducted on nano/micro particles and hydrogels for cardiac related ailments.

**Clinical trials**	**Therapeutics**	**Type**	**Clinical trials ID**
Magnetic Nanoparticles System in Acute Coronary Syndrome	Magnetic nanoparticles for diagnostics	Nanoparticles	(NCT02226523)
Plasmonic Nanophotothermal Therapy of Atherosclerosis (NANOM-FIM)	Bioengineered cardiac patch to regress atherosclerosis	Nanoparticles	(NCT01270139)
Treatment of Patients With Atherosclerotic Disease With Methotrexate-associated to LDL Like Nanoparticles	LDL binding Methotexate nanoparticles	Nanoparticles	(NCT04616872)
Microvascular Reperfusion Utilizing Sonothrombolysis in Acute Myocardial Infarction (MRUSMI TRIAL) (MRUSMI)	Sonothrombolysis to break up blood clots in arteries	Microspheres	(NCT02170103)
Contrast ICE for myocardial Scar in VT Ablations	Diagnostic echocardiography to visualize scar tissue formation	Microspheres	(NCT03212326)
Autologous Human Cardiac-Derived Stem Cell to Treat Ischemic cardiomyopathy (ALCADIA)	Human cardiac derived stem cells and bFgF for treating refractory heart failure	Hydrogel	(NCT00981006)
First in Man Study of Implantable Alginate Hydrogel	Implantable Alginate Hydrogel for left ventricle reconstruction	Hydrogel	(NCT04781660)
COVADIS Pilot Trial: COseal in Ventricular Assist Devices (COVADIS)	Surgical hydrogel used to prevent cardiac adhesions	Hydrogel	(NCT01244321)

Accurate and timely diagnosis of acute coronary syndrome (ACS) is of great clinical importance. Therefore, magnetic NPs surface-functionalized with bio-probes was developed for rapid detection of early ACS diagnostics markers (NCT02226523). The principle behind the technology is to assess the magnetic oscillation of the NPs when exposed to alternating current, as these NPs interact with biomarker protein from the blood samples retrieved from patients; it undergoes aggregation resulting in the reduction of magnetic oscillation events known as immunomagnetic reduction (107). Thus, this technique offers real-time, rapid detection of clinically relevant biomarkers that can aid in early MI diagnosis.

The formation of atherosclerotic plaque in the coronary arteries is considered a primary predisposing factor for MI. Nanoparticle-based therapies have been developed and tested for the eradication of atherosclerotic plaque. Silica-gold and Fe_3_O_4_ magnetic NPs have been used for photothermal ablation of atherosclerotic plaque both non-invasively and in a minimally invasive manner (NCT01270139). The NIR light used exhibits deep tissue penetration making it possible for irradiation from outside the body resulting in a remarkable reduction in atherosclerotic plaque compared with controls without adverse events. This has emerged as a newer paradigm in treating atherosclerotic plaque (108, 109). Sonothrombolysis is another new therapy that has also been explored to break down thrombus using ultrasound combined with perflutren lipid microspheres (NCT02170103). The ultrasound frequency within the range of 1-1.7 MHz has significantly reduced infarct size and provided regional functional recovery (110). It has also been demonstrated that perflutren lipid microspheres can be used for contrast imaging to identify foci responsible for ventricle tachycardia. The current clinical approach for treating ventricular tachycardia is by catheter-based ablation, which requires electrical mapping of ventricles to locate the foci; ultrasound-based tissue imaging could further identify potential arrhythmogenic foci within scar (NCT03212326). Therefore, researchers are using these lipid-based microspheres that could aid in the accurate diagnosis of arrhythmias.

The localized inflammatory response is one of the main driving factors in the formation of atherosclerotic plaque. Treatment with anti-inflammatory agents has shown some degree of inhibition in plaque progression (111). A lipid-based nanosystem mimicking LDL encapsulating methotrexate (as an anti-inflammatory agent, NCT04616872) has been tested. Clinically higher doses of methotrexate may result in severe systemic adverse events. However, by using nanoparticle-based targeted delivery, inflammatory lesions can be treated without developing off-target toxicities. Animal studies have shown the ability to inhibit pro-inflammatory cytokines while upregulating anti-inflammatory cytokines with a significant reduction in scar size (112).

Hydrogel entrapping bFGF has also been investigated in clinical studies. An implantable crosslinked gelatin hydrogel sheet was fabricated and investigated for improving the engraftment and survivability of injected stem cells (NCT00981006). The autologous cardiac stem cells were implanted in bFGF gelatin hydrogel sheets in patients with severe LV dysfunction. The results demonstrated a decrease in scarring, and a significant enhancement in LV functioning within 6 months of implantation, and improved exercise tolerance. Moreover, the clinical data suggest that this biotherapy can be safely administered to patients (113).

Similarly, alginate-based hydrogels are used as a support material and investigated to treat ischemic and non-ischemic cardiomyopathy (NCT04781660). Lastly, a polyethylene glycol-based *in situ* crosslinked hydrogel (CoSeal®) is under investigation for its applicability to prevent tissue inflammatory response caused by left ventricular assist device (LVAD). This hydrogel application is anticipated to avoid the inflammatory response and enable surgeons to remove VADs with minimal surgical interventions (NCT01244321) more easily.

The above-discussed clinical studies have demonstrated the efficacy of biomaterials. Their use as NPs, MPs, or hydrogels shows promise in treating CVD and can be employed in cardiac repair; however, there is a need for further investigation, innovation, and exploration of emerging smart biomaterials to impart therapeutic benefits.

## Conclusion

Overall, the discovery of newer functionalized polymers provides an excellent potential for achieving sustained delivery of bioactive growth factors in treating the damaged heart. The availability of advanced techniques at our disposal for fabricating nanoparticles and microparticles has facilitated the ease of upscaling at the industrial level. These have also made it possible to control the size from nano to micro range with a great degree of spatio-temporal control, directly affecting drug release rate. In addition, the ever-growing field of biomaterials has demonstrated the possibility that smart hydrogels with sophisticated properties like electroconductivity have further expanded the spectrum of their application in the cardiac field. Another potential future application of these innovative biocompatible polymers could be localized immune-modulation, which can offer therapeutic potential in alleviating adverse immune reactions and aid in healing the damaged heart. The localized availability of therapeutic bioactive molecules has shown promising results in preclinical studies. Therefore, these therapies can further be explored in a clinical setting to facilitate the translation from bench to bedside.

## Author Contributions

SBA and MK: conceptualization and design. SBA, SA, DS, ZN, NP, HW, KB, WZ, NS, and MK: wrote and reviewed the manuscript. The input from all the authors was incorporated in finalizing the manuscript draft.

## Conflict of Interest

The authors declare that the research was conducted in the absence of any commercial or financial relationships that could be construed as a potential conflict of interest.

## Publisher's Note

All claims expressed in this article are solely those of the authors and do not necessarily represent those of their affiliated organizations, or those of the publisher, the editors and the reviewers. Any product that may be evaluated in this article, or claim that may be made by its manufacturer, is not guaranteed or endorsed by the publisher.

## Abbreviations

ACS: acute coronary syndrome
ADM-2: adrenomedullin-2
bFGF: basic fibroblast growth factor
BIO: 6-Bromoindirubin-3-oxime
BMSCs: bone marrow mesenchymal stem cells
CABG: coronary artery bypass grafting
CAT: catalase
CVD: cardiovascular diseases
ECM: extracellular matrix
EV: extracellular vesicles
FGF1: fibroblast growth factor-1
GMA: glycidyl methacrylate
hESC-CMs: human embryonic stem cell-derived cardiomyocytes
hESC-ECs: human embryonic stem cell-derived endothelial cells
HGF: hepatocyte growth factor
IGF-1: insulin-like growth factor
IL-1: interleukin-1
IL-1β: interleukin-1β
IV: intravenous
LV: left ventricular
LVAD: left ventricular assist device
MI: myocardial infarction
NPGs: nanoporous gels
NPs: nanoparticles
NRG-1: neuregulin-1
PDGF: platelet-derived growth factor
PEC: polyelectrolyte complex
PEG: polyethylene glycol
PEGDA: polyethylene glycol di acrylate
PEI: polyethyleneimnine
PFBT: poly(9,9-dioctylfluorene-alt-benzothiadiazole)
PLA: polylactic acid
PLGA: poly(lactic-co-glycolic acid)
POD: peroxidase
PVA: polyvinyl alcohol
ROS: reactive oxygen species
SDF-1α: stromal cell-derived factor 1
Shh: sonic hedgehog
SOD: superoxide dismutase
TIMP-3: tissue inhibitor of metalloproteinases-3 inhibitor
TPP: triphenylphospine
Tβ4: thymosin beta 4
VEGF: vascular endothelial growth factor.
